# Hodgkin Lymphoma—The Spectrum from Diagnostics to Molecular Science, Movement and Current Treatment Approaches

**DOI:** 10.3390/cancers15102726

**Published:** 2023-05-12

**Authors:** Sylvia Hartmann

**Affiliations:** Dr. Senckenberg Department of Pathology, Goethe University, 60590 Frankfurt, Germany; s.hartmann@em.uni-frankfurt.de

A total of fourteen papers on Hodgkin lymphoma (HL) are published within this Special Issue, including six reviews, seven original articles and one commentary. The spectrum ranges from papers covering basic molecular research results and translational studies to reviews on therapeutic options in special settings of HL.

Firstly, two reviews summarise the recent diagnostic standards of the diagnosis of classic and nodular lymphocyte predominant HL. The review by Bosch-Schips et al. [1] deals with the diagnostic criteria of classic HL and delineates it from differential diagnoses with overlapping morphological features. In particular, the differential diagnoses of primary mediastinal B-cell lymphoma, mediastinal grey zone lymphoma, EBV-associated B-cell lymphoproliferative diseases with Hodgkin-like features, nodular lymphocyte predominant HL and classic HL with expression of T-cell markers are featured.

The review by Younes et al. [2] focuses on the diagnostic boundaries between nodular lymphocyte predominant HL (NLPHL) and differential diagnoses such as diffuse large B-cell lymphoma, T-cell/histiocyte rich large B-cell lymphoma and mature T-cell lymphomas, and also non-neoplastic conditions such as progressively transformed germinal centres. It also discusses emerging concepts in NLPHL.

After a diagnosis of HL has been established, it is important that the best treatment options can be offered to the patient. In this respect, the review by Follows and Santarsieri [3] summarises different approaches to combined modality treatment with regard to different stages and favourable or unfavourable prognostic factors. They particularly review approaches that aim to reduce toxicity and improve efficacy, since a high rate of disease-free survival is achieved by most strategies. They discuss the results from different clinical study groups with and without interim PET (iPET) results for decision making. With regard to iPET, the study by Zheng et al. [4] re-evaluated the role of iPET in a retrospective patient cohort that was treated with six cycles of ABVD irrespective of the iPET results. Different options of treatment choices in advanced stages of cHL are discussed in the review by Vellemans and André [5]. They also discuss treatment adaptation after iPET and novel treatment strategies like the antibody–toxin conjugate Brentuximab-Vedotin and Checkpoint inhibition. The roles of iPET, as well as different strategies for how to assess iPET in a residual mass, such as the Deauville Score or Standardised Uptake Volume, are discussed in a review by Gallamini et al. [6]. They also present recent challenges such as interpreting a residual mass during Checkpoint Inhibitor therapy, which reveals new aspects related to the activity of the drug, but also related to the fact that there are no defined cycles like in a standard ABVD regimen.

The review by Navarro et al. [7] deals with a specific Hodgkin patient group: those that have a concomitant HIV infection. People living with HIV (PLWH) have a 5- to 26-fold increased risk of developing a cHL, and are thus an important patient subgroup. Although nowadays the treatment and outcome of PLWH is comparable with the general population, it is necessary to be aware of this group of cHL patients in the diagnostic setting and also in the management of anti-retroviral therapy in addition to lymphoma-specific therapy.

Finally, the commentary on the use of Pegfilgrastim in HL by Cerchione et al. [8] highlights not only the importance of lymphoma-specific treatment, but also the management of side effects, since therapy-induced neutropenia can be a life-threatening event in HL patients.

The other part of this Special Issue contains six original articles covering basic research and translational studies on HL.

The study by Gusak et al. [9] investigates a very up-to-date topic dealing with the composition of the microenvironment and changes during checkpoint inhibitor therapy by immunohistochemistry. In contrast to what was observed in the pre-checkpoint inhibition era [10], they reveal that a low number of macrophages in the HL microenvironment was associated with inferior PFS during Nivolumab treatment. Another study focusing on microenvironment is the paper by Lamaison et al. [11], which aimed to establish a signature identifying patients at risk for relapse. Since patients with early HL relapses still have an unfavourable prognosis, the study aim is an important issue in the field of HL. In contrast to the general observation that HL subtypes are not prognostic under current state-of-the-art stage-adapted therapy, the study by Lamaison et al. suggests a role of the HL subtype in the identification of a patient group at risk for relapse. Paczkowska et al. [12] contributed to this issue by identifying miRNAs that affect the activity of members of the NF-kappaB pathway and B-cell receptor signalling, and thus further explain the unusual tumour cell phenotype that is observed in the Hodgkin–Reed–Sternberg cells of cHL. The study by Jiang et al. [13] identified two so-far-unnoticed gene loci, ERAP1 and ERAP2, that interact with HLA class I and further point to the importance of antigen presentation in the pathogenesis of HL. The study by Hartmann et al. [14] unveils differences in the T-cell movement patterns when comparing HL with other B-cell lymphomas. The scanning properties of T-cell movement in HL also point to a role of antigen presentation between tumour cells and T-cells in HL.

Finally, the paper by Agrusa et al. [15] deals with the pre-therapy plasma proteome in paediatric HL. They identify unique cytokines/chemokines associated with high-risk disease (IL-10, TNF-α, IFN-γ, IL-8) and slow early response (CCL13, IFN-λ1, IL-8). They identify TNFSF10 to be predictive for relapses.

In summary, this Special Issue covers the various facets of HL from the different perspectives of diagnostic, therapeutic and molecular points of view, and sheds light on this unique and particularly interesting type of lymphoma.
